# Stages in the psychological resolution of schizophrenia

**DOI:** 10.3389/fpsyg.2015.00086

**Published:** 2015-03-04

**Authors:** Gillian R. M. Steggles

**Affiliations:** Centre for Psychoanalytic Studies, University of Essex, Colchester, UK

**Keywords:** schizophrenia, recovery, psychoanalytic psychotherapy, motivation, PPCC model

## Abstract

From the work of Dr. Michael Robbins in Massachusetts, USA, it is known that nine schizophrenic patients out of a series of 18, and a further schizophrenic patient, treated by him achieved positive outcomes using psychoanalytic methodology. Four of these had strikingly successful outcomes, for example completing their treatment without a need for further medication, and also becoming happily married or graduating at university. This paper aims to illustrate the stages identified by him through which this can be accomplished. Dr. Robbins’ Stages of Psychological Therapy of Schizophrenia are compared with Dr. Steggles’ detailed case study of a patient’s recovery from schizoaffective disorder. These two data sources are juxtaposed and compared. Dr. Robbins’ therapeutic stages are found to parallel exactly Dr. Steggles’ findings from her case study, which she summarized as her psychodynamic pentapointed cognitive construct (PPCC) model of her schizoaffective patient’s experience. Psychological therapy of schizophrenia is still in its early stages of development. However, Dr. Robbins’ psychoanalytic psychotherapeutic technique has given rise to positive outcomes in 10 of the 19 patients he treated, that is, his series of 18 patients together with a further patient; these 19 patients he gave full psychological treatment, i.e., usually four sessions per week. The Stages he identifies in his therapeutic process match perfectly the stages Dr. Steggles identified in her own patient’s healing mind. Not all schizophrenic patients are likely to be able to benefit from this psychological therapy. Females seem to be better able than males to respond to the treatment, and motivation is necessary for a successful outcome. It is not known how to identify precisely those patients who will be successful. But those patients who do benefit may counterbalance by their economic activity the healthcare costs of those who do not recover, as well as achieving benefit from their human suffering. Many of the other groups of patients suffering from schizophrenia can be helped by engaging with a clinician for social skills or family therapy, and where appropriate this should always be done.

## METHOD

**FIGURE 1:**
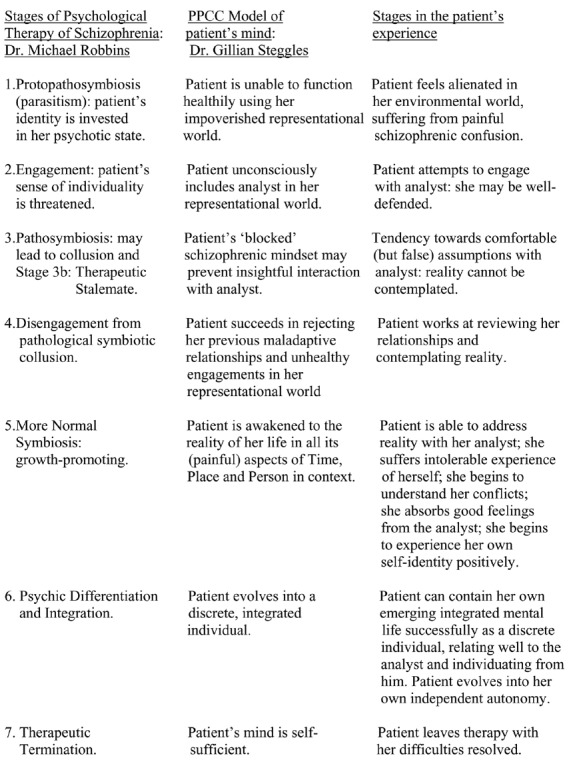
**Stages in the psychological resolution of schizophrenia**.

Dr. Robbins’ experience in the psychological therapy of schizophrenic patients led him to outline (Robbins, 1993, p. 259) a staging of its process (see Figure 1) which applied to them all as a group of similarly managed cases. The staging fell into two sections. The first phase, Stages 1 to 3b, describes the patient, hindered by her schizophrenic perception and experience, as being unable to relate to the analyst except unrealistically. The second phase, Stages 4 to 7, involves a steep learning curve for the patient in light of reality after engaging healthily with the analyst; the patient’s characteristics become exposed to her own scrutiny and then to her own capacity for personal adjustment and acceptance.

Nine of Dr. Robbins’ series of 18 patients were unsuccessfully treated, but their results are included in the Staging sequence he compiled, through reaching therapeutic stalemate at Stage 3b. Most of his patients required prolonged hospitalization, up to several years at a stretch; he worked with some for 15 years or more. They all satisfied the DSM-III-R criteria for schizophrenia. The patients’ need for treatment was in all instances precipitated by major failures of adjustment at work, school or relationship. Treatment efforts for four of his nine unsuccessfully treated patients failed after less than a year. Dr. Robbins found that patients with “significant affect” and with positive rather than negative schizophrenic symptoms had better results from treatment. Of the nine patients with positive results in his series, six had very successful results, two had continuing significant problem areas although not specifically involving psychosis, and one patient’s treatment was incomplete. A further patient treated after his series of 18 patients proved to demonstrate one of his most successful outcomes.

Dr. Steggles made a very detailed, long term study as a clinical researcher of a young schizoaffectively disordered student, and from it derived her psychodynamic pentapointed cognitive construct (PPCC) model of the functional psychoses (Steggles, 2012). The PPCC model describes the evolution of the student’s mind, from its original schizoaffective state where it depended for its survival on the student’s representational world, impoverished and painful though this was, to independent autonomy. Sandler and Rosenblatt’s (1962) representational world is a partly conscious, partly unconscious representation of significant features of the individual’s external environment which have been meaningful and important to her in her life thus far. Feelings and meanings are attached to the representational world, which acts as a guide to future experiences.

Dr. Steggles’ patient made a small study of her mind by examining what came into it when she “stilled” it in the manner of a Buddhist meditation technique. Twenty-nine ideas entered her mind, in five naturally occurring groups, which she wrote down, and then arranged in a pentapointed shape. Examination of these data showed that these ideas formed her representational world, the aspects of her lifelong experience which were especially meaningful for her. The student highly valued these contents of her mind. Dr. Steggles elaborated her PPCC theory of the functional psychoses from the student’s small study. This theory is, in particular, a description of how a schizoaffective mind changes as the patient’s health improves toward mental health. Dr. Steggles then drew up as parallel processes this sequence of the schizoaffective patient’s experience and Dr. Robbins’ therapeutic Stages.

## RESULTS

The phases of Dr. Steggles’ PPCC model of the patient’s perspective were found to coincide precisely with the Stages that Dr. Robbins observed in his patients as a clinician.

Initially, the student was dependent upon her representational world as being the lucid and recognizable substance of the manifest contents of her mind. Subsequently, she found herself unable to think clearly due to her illness. Then she became capable of containing her mind’s more fluid contents independently (a 3-dimensional form of her mind which could contain memories); and finally she achieved independent autonomy (a smooth, self-sufficient sphere). These stages of the PPCC model involve firstly, her being trapped inside her impoverished representational world (Dr. Robbins’ Stage 1) until she engages with the analyst as a part of this unhealthy world (Stage 2); subsequently, a schizophrenic stalemate becomes established in the patient’s mind which coincides with Dr. Robbins’ therapeutic stalemate (Stage 3); then, after overcoming this stultification phase (Stage 4), the patient’s orienting mind actively identifies her lifetime’s remembered events healthily in time, place and person, which coincides with Dr. Robbins’ stage of therapeutic symbiosis (Stage 5); and eventually the patient’s independent autonomy coincides with Dr. Robbins’ psychic differentiation and integration, and therapeutic termination (Stages 6 and 7). In this way Dr. Steggles’ understanding of her patient can be summarized; and the steps of her patient’s experiential progress can be seen to parallel Dr. Robbins’ therapeutic Stages observable by the clinician (Figure 1). The PPCC’s geometric designs help the processes of the patient’s healing mind to be recognized and grasped, but are not necessary for understanding them which may be attained equally well from Figure 1.

Both Dr. Robbins’ therapeutic stages and the PPCC model are psychoanalytically based. Dr. Robbins’ technique “depends upon attitudes and techniques of therapist self-analysis” (Robbins, 1993, p. 266), and the PPCC model is a psychoanalytic understanding of a patient suffering from a psychotic disorder (Steggles, 2012). Dr. Robbins’ schizophrenic patients and Dr. Steggles’ schizoaffective patient all used antipsychotic medication, including commonly trifluoperazine; chlorpromazine was found to be too sedative. So the stages documented for these two sources of clinical material involve both Psychiatry and Psychoanalysis, as approaches to thinking about the psychological processes through which schizophrenic patients can be helped.

## DISCUSSION

The mode of action of psychoanalysis seems to present so much doubt in the minds of some that the possibility of its efficacy is denied. It is true that some aspects of it are not fully understood, and also that every experience of psychoanalysis is different and specific both to the analysand and to the analyst. It is a broad and complex field.

However, there is so much in common among successful psychoanalyses that it is worth trying to identify which aspects have contributed to these successes and which are the salient worthwhile features. In a successful analysis the analyst is gentle, emotionally empathic, perceptive, insightful, attentive, generally cheerful, and dependable. The patient needs to be motivated, intelligent, patient, determined, forgiving of themselves and others including the analyst, capable of insight and of internal, personal learning, and able to pick herself up after painful realizations during the sessions and to continue in spite of these.

Difficulties such as misery, despair, resentment, hostility, boredom, confusion, fears and anxieties, and others specific to the patient may be encountered. But psychoanalysis can be a very effective treatment for alleviating deep-seated psychological problems, while it is also true that one or more of these difficulties mentioned may prove insurmountable; and it may be the case that those individuals who level the fiercest attacks on psychoanalysis have had a bad experience of it through one of the many ways that it can go wrong either for patient or analyst. However, detailing the stages demonstrates how a psychoanalysis can be kept going through the faith of identifying what can be expected at each stage. Herbert Rosenfeld’s book “Impasse and Interpretation,” published in 1987 after his death (Rosenfeld, 1987), illustrates his research on the therapeutic impasse. He emphasizes the analysts role in the occurrence of impasse, but Dr. Robbins has clearly shown within his own clinical practice that its threat can be mastered.

The efficacy of a psychoanalysis is dependent upon a patient’s motivation. Some patients seem to possess it, and others not. It is what lies behind her attending her appointment day after day at her analyst’s consulting-room even when she feels most miserable and despairing. This is where the analyst’s gentleness, general cheerfulness, empathy and emotional generosity are so important for continuity of the treatment. The patient should feel that it has been so worthwhile for her to have made the effort to attend her appointment, once she is in the session, due to the analyst’s understanding and empathy, and all of this built on the patient’s desire to be well.

The schizophrenic patient’s anticipatory or extrinsic motivation, which governs their desire for rewards, is distinct from their intrinsic motivation, which stimulates activity for its own sake without any other objective likelihood of a reward (Barch et al., 2008). Schizophrenic patients’ extrinsic motivation appears to be adversely affected by their illness: they cannot evaluate reward and punishment (Kim et al., 2012). By contrast, motivational deficits in schizophrenia appear not to reflect impairments in intrinsic motivation (Barch et al., 2008). Thus schizophrenic patients sometimes have sufficient intrinsic motivation to keep going with their therapy.

The patient’s motivation is one of her key assets. Two others which are also essential are her intelligence and ability to evaluate what her analyst says to her in his interpretations, and also her ability to self-scrutinize, or insight. At first her insight may be quite limited. However, through her motivation and using her intelligence, her insight may accumulate; this is, after all, the main goal of a psychoanalysis. And necessary after gaining insight is the patient’s application of this self-knowledge in practical terms to the reality of her life. A schizophrenic patient has a particularly difficult time because her mind is so difficult to relate to, with its “alien” tendencies to function very differently from how the patient would wish, such as hallucinating. Medication is a great help in calming these alien tendencies, which may or may not be due specifically to disordered dopamine physiology. The medication assists the schizophrenic patient’s relation to her own mind, together with the analyst’s influence, until she becomes better at using her mind; if the patient can learn to trust the medication as well as the analyst then she has an excellent opportunity for thinking through and enacting improvements to her circumstantial and relational difficulties so that she actually feels better due to these practical changes. Fresh insights about her relationships strengthen her knowledge about herself. Self-esteem now has a justified basis upon which to improve. Thus the patient moves through the stages of her psychoanalysis gradually feeling better on an entirely realistic basis: she actually is getting better. This improvement can be measured using self-report methods (Cavelti et al., 2012). Self-assessment has led to a substantial improvement in the understanding of recovery from its traditional basis in simply the disappearance of symptoms, functional improvement, and reduced use of medical health services. So unquestionably, patient improvement can be demonstrated.

Psychodynamic psychotherapy has been shown to be very effective in the long term as a treatment method in regulating emotional disorders, exploration of distressing thoughts and feelings and fantasy life, identifying recurring themes and patterns, and discussion of past experience and interpersonal relations (Shedler, 2010). Dr. Robbins has shown how it can be applied to the treatment of schizophrenia.

The therapeutic pathway identified by Dr. Robbins and described in this paper as being that of his 19 schizophrenic patients may provide a basis for further efforts to bring patients who feel entirely lost inside themselves into the world of the living. These patients know clearly that something is very wrong with themselves; and this can be a fearful realization, especially when no help is apparently available, or else only pills or injections.

These patients need and deserve effective therapy as emphatically as patients who need open heart surgery, complex and expensive prostheses or mobility chairs. These treatments are visible, concrete and easily conceptualized. Psychological treatments are invisible, abstract and can be very difficult to conceptualize. But this is absolutely no reason why those who allocate funds should, in practice, overlook the need for them.

Carefully allocating precious psychological medicine funds to schizophrenic patients who are motivated, intelligent, persevering and determined can be an excellent investment into lives initially blighted. The analyst needs to be skilled and experienced, learning from the paths of those who have gone before him. He needs to be psychiatrically trained in order to be able to work with psychotic patients. It has been said that psychoanalyzing a psychotic patient requires a mental state examination every 5 min. Psychoanalysts need confidence, courage and the support of skilled and well-staffed psychodynamic hospital units, which can care for these vulnerable patients while they do their best to work through the experiences of their treatment.

It seems that neither cognitive behaviour therapy (CBT) nor psychoanalytic therapy gives consistent results in alleviating schizophrenia. But the usefulness of psychoanalytic psychotherapy lies in its selective, very effective application for those patients who are alert enough to be aware of themselves as being assailed by their illness when they could be doing other, useful things with their life; patients who struggle but in vain to fulfill their own wishes. These are the patients who may have strong reserves of energy, resource and motivation to continue therapy until they emerge successfully in their life and liberation from schizophrenia.

## CONCLUSION

Dr. Michael Robbins’ Stages of Psychological Therapy of Schizophrenia, developed after his work with at least 19 schizophrenic patients, are corroborated by Dr. Steggles’ detailed case study research on her schizoaffective patient. A positive outcome was achieved with 10 of Dr. Robbins’ 19 patients, with female benefiting patients outnumbering males. A significant number of patients were initially assessed for up to 6 months who were not considered able or suitable to continue with fulltime therapy; and, as has been seen, 9 of those 19 who were treated fulltime by Dr. Robbins failed to progress to therapeutic termination. So, much of the success of this therapy lies in careful patient selection after a 6 months assessment; and in a prevalence of positive over negative symptoms; and in the presence of “significant affect.” Taking medication is not in itself a contraindication to therapy. Those considered not suitable for psychoanalytic psychotherapy should be offered benefit from all other available therapies including CBT, family therapy, and social skills therapy together with medication.

The opportunity of mental health, manifesting initiative, creativity, versatility, happiness, peace and global effectiveness, is what psychoanalytic psychotherapy of the kind provided by Dr. Robbins to his patients can lead to, and has achieved. His practice numbers indicate that 10 of his 19 fully treated cases reached this result or near it. Applied by others, his approach may be less successful, possibly due to less strong personal connection to the patients and less good communication of optimism or confidence as a therapeutic tool. But he has identified as an overview the Stages of treatment response among his schizophrenic patients, together with its explanation, which has been independently corroborated by another detailed case study. Surely our task is to attempt to replicate and follow his achievement for the patients who can benefit from it, while continuing to do everything we can for those who are less fortunate.

### Conflict of Interest Statement

The author declares that the research was conducted in the absence of any commercial or financial relationships that could be construed as a potential conflict of interest.
